# Multi-centre comparison between device-independent web-browser perimetry (Melbourne Rapid Fields-web) and SITA-Faster for glaucoma

**DOI:** 10.3389/fopht.2025.1485950

**Published:** 2025-02-06

**Authors:** Joyce Tiang, Algis J. Vingrys, Sarah Lin, Selwyn M. Prea, Adam Ahmed Moktar, Allan Bank, Ashish Agar, Yu Xiang George Kong

**Affiliations:** ^1^ Department of Ophthalmology, Royal Victorian Eye and Ear Hospital, East Melbourne, VIC, Australia; ^2^ Department of Optometry and Vision Sciences, Faculty of Medicine, Dentistry and Health Sciences, The University of Melbourne, Melbourne, VIC, Australia; ^3^ Department of Ophthalmology, Centre for Eye Research Australia, East Melbourne, VIC, Australia; ^4^ Department of Ophthalmology, The University of New South Wales, Sydney, NSW, Australia; ^5^ Department of Ophthalmology, The Dubbo Eye Centre, Dubbo, NSW, Australia; ^6^ Department of Ophthalmology, Prince of Wales Hospital, Sydney, NSW, Australia; ^7^ Department of Ophthalmology, Department of Surgery, The University of Melbourne, Melbourne, VIC, Australia; ^8^ Department of Ophthalmology, Mt Waverley Eye Surgeons, Melbourne, VIC, Australia

**Keywords:** visual field, rural medicine care, portable perimeter, glaucoma, glaucoma & fields, Melbourne Rapid Field (MRF), SITA faster, diagnostic accuracy (either seperate or as a replacement of diagnostic performance)

## Abstract

**Purpose:**

Visual field testing is important for glaucoma diagnosis and management, but access to standard automated perimetry can be limited in some areas due to cost or access. Melbourne Rapid Fields-web (MRF-web) perimeter is designed to address these limitations by allowing perimetry testing on the flat screen of your personal computer.

**Methods:**

This study is a retrospective, cross-sectional study involving two locations in Australia, one in metropolitan Melbourne and one in rural Dubbo NSW. 232 patients with stable glaucoma, glaucoma suspect or normal eyes were tested with MRF-web and outcomes were compared to the most recent Humphrey Field Analyzer (HFA) 24-2 SITA Faster test. Outcomes were compared by Deming regressions, Intraclass Correlation Coefficients (ICC) and Bland-Altman methods.

**Results:**

Patient age ranged from 21 to 92 (average 66.3, SD 16.1). Bland-Altman found a bias of -0.50dB for Mean Deviation (MD) between the two tests, with 95% Limits of Agreement (LoA) of -6.80dB to 5.80dB. Pattern Deviation (PD) had a bias of -0.58dB with 95% LoA of -5.60dB to 4.40dB. High concordance was found for MD and PD, with ICCs of 0.87 and 0.73. No significant differences were found in false positive and fixation loss rates. Test time was approximately one minute longer for MRF-web compared to SITA-Faster. Area Under the Curve of MRF and HFA are similar indicating comparable diagnostic capacity.

**Conclusion:**

MRF-web produces outcomes comparable to HFA SITA-Faster. Its portability and cost-effectiveness suggest suitability as an alternative method for visual field testing where a standard perimeter is not easily accessible.

## 1 Introduction

Glaucoma is a chronic and progressive ocular condition that is a leading cause of irreversible blindness worldwide (1, 2), both in rural and metropolitan areas (3). It is characterized by the loss of retinal ganglion cells, which translates to corresponding visual field defects (4), therefore, visual field testing is important for diagnosis and management of glaucoma (5). It is also a critical tool for determining the stage of the disease, prognosis, and efficacy of treatment being offered to patients (6, 7). Standard automated perimetry (SAP) remains the preferred method for evaluating a patient’s visual field. However, most modern SAPs are not easily portable, requiring trained professionals to set up and guide patients throughout the assessment and are expensive (8). Furthermore, as visual field loss in glaucoma is often asymptomatic (9), it is important to have accessible visual field testing methods (10, 11).

To improve the accessibility and portability of visual field testing, various technologies have been introduced including tablet-based, computer screen-based and goggle virtual reality-based technologies (7). Melbourne Rapid Fields (MRF) is a portable perimetry software (previously available as an application on iPad) that has been validated against Humphrey Field Analyzer (HFA) and has good intrasession test-retest repeatability (8, 12–15), even with variations in ambient light levels, blur and viewing distance (13). In 2020 MRF was translated to a device-independent web-browser application to facilitate remote home testing during COVID lockdowns. Recent work by Harris et al. has evaluated the web-based version of the MRF (MRF-web) in 40 young healthy optometry students and found good concordance (average test-retest difference in both eyes = 0.13 dB) between the test performed in-clinic compared to at-home on that evening (16).

This study will examine the function and the application of the MRF-web by assessing the level of agreement between MRF-web and the HFA 24-2 Swedish Interactive Thresholding Algorithm (SITA)-Faster algorithm (HFA SITAfr) involving patients on a spectrum of glaucoma severity from both metropolitan Melbourne and Rural NSW.

## 2 Methods

### 2.1 Participants

A multi-centred cross-sectional observational study was conducted on 232 participants who presented for routine review to one of two locations, Mount Waverley Eye Surgeons in Melbourne, Victoria, Australia and Sydney Eye Care in Dubbo, a regional city in New South Wales, Australia. All testing reported in this study was performed in a clinical setting at both locations.

All patients included were categorised into three groups as either having stable glaucoma, glaucoma suspects or normal after a comprehensive eye examination by a glaucoma specialist (AA, AB, GK). This included a general and ocular history and medications, visual field outcome using the SITA Faster 24-2 test (Humphrey Field Analyser, HFA), gonioscopy, fundus examination of the optic nerve head, posterior pole and peripheral retina with slit-lamp biomicroscopy, optical coherence tomography, and optic disc photography. The SITA Faster (SITAfr) strategy was chosen as it aligned with the operational needs of the participating clinics, where optimizing patient flow while maintaining the clinical relevance of visual field data was a priority. Additionally, its widespread adoption across clinics made it an appropriate choice for comparing the MRF with the HFA SITA Faster, thereby enhancing the study’s clinical applicability. However, it is worth noting that the HFA SITA Standard strategy is nevertheless more reliable than SITAfr for detecting subtle visual field changes (17). The severity grading of glaucoma is categorised depending on the MD outcomes from the SITAfr utilising a modified Hodapp-Parrish-Anderson classification (18): normal (MD>-2), mild (-2<MD>-6), moderate (MD<-6), advanced (MD<-12) and severe (MD<-20).

Participants met the inclusion criteria if at least one eye had a visual acuity of 6/12 (20/40) or better. Eyes with coexisting conditions such as diabetic retinopathy and age-related macular degeneration were also included provided that it met the inclusion criteria of having visual acuity of at least 6/12. The right eye of the participants was selected for the study, if the right eye did not meet the inclusion criteria, the left eye was used. Participants were excluded if they had intraocular surgery 6 months prior to any visual fields. Lens status was not a criterion for exclusion, although any lens opacity could not limit visual acuity to worse than the 6/12 (20/40) inclusion criterion. Of the 232 patients included in the study, 94 patients (41%) completed HFA SITAfr before MRF-web, 63 patients (27%) completed the MRF-web before HFA SITAfr, and 75 patients (32%) completed both tests on the same day.

The study was undertaken with the approval of the RANZCO (Royal Australian and New Zealand College of Ophthalmologists Ethics Committee (Study 143.22) and was conducted in accordance with the tenets of the Declaration of Helsinki. All subjects gave informed consent for participation.

### 2.2 Testing procedure

The MRF application has been described in detail in our previous studies and a 24-2 test grid was used in the current study (8, 13). Threshold is achieved by a rapid three-presentation neighbourhood ZEST protocol that yields seven discrete steps over a 0 to 30 dB range (0, 6, 12, 17, 22, 26, 30 dB) (13). One reason for using neighbourhood logic is to recheck locations removed from the local neighbourhood expectation. Spots are shown on a 5 cd.m^-2^ background and increase in size in peripheral locations from about a Goldman size II in the fovea to Goldman size V beyond 30 degrees (19). The purpose of spot-size scaling is to allow for the geometry of the screen and to return constant thresholds and reduced variability at peripheral locations (19, 20). The patient’s response to the presence of a stimulus is polled by touching the spacebar on the keyboard. A modified 24-2 grid was used with the MRF which includes the same positions as the HFA SITA-Faster (SITAfr) 24-2 Grid plus 4 additional foveal points at 0.75 degrees eccentricity.

All testing was undertaken by a clinical assistant using MRF-web software. MRF-web is a browser version of the original MRF software (8, 15) that has been designed to operate on any laptop or tablet provided the screen has a diagonal extent of at least 9.7 inches (246 mm). In this study, we utilised an LG desktop computer with a 27-inch screen at the Melbourne location and a 13-inch Apple MacBook Air for testing in the Dubbo centre. The devices access the MRF-web browser application with Google Chrome. The MRF software implements a calibration step that identifies pixel density and size of the screen when it is first used by having the user move two mires to match the short side of a standard credit card (54 mm). For the 27-inch screen, the software utilises a viewing distance of 50 cm, and for the 13-inch screen, the viewing distance is 33 cm. The software requests the user to set the brightness of the screen to maximum. All calibration and brightness adjustments were performed by the clinical assistant prior to testing patients. The clinical assistant also detailed the test procedure to the patient and voice prompts were given in English by the device during the test to guide the user through the test. Additionally, the Webcam of the device was used for facial tracking to continuously monitor the viewing distance throughout the test by comparing the present size of the face to the size set at the desired viewing distance at the start of the test (21).

Participants were introduced to the test procedure with a 30-second practice run. The clinical assistant ensured the test room was darkened, and no glare was evident on the screen prior to each test. Testing was performed with natural pupils. Additionally, patients were asked to wear their habitual reading glasses as required for normal near viewing.

Participants wore their habitual reading glasses as needed by their presbyopia or ametropia. The fellow eye was patched, and software analysis of the face ensured that this was so.

### 2.3 Statistical analysis

Outcomes from the MRF-web assessment were compared with those found on the HFA 24-2 SITAfr algorithm. All analyses were done using SPSS (SPSS version 29.0.1.0; SPSS Inc., Chicago, IL) or GraphPad Prism v10.1.1 for Mac (GraphPad Software, Boston, MA USA, https://www.graphpad.com). Interclass coefficients (ICC) report concordance for MD and PSD when comparing HFA and MRF. A Bland-Altman analysis was undertaken to identify bias, and 95% Limits of Agreement (LoA) in comparing the HFA and MRF outcomes. Either t-test or repeated measure analysis of variance (ANOVA) with a significance level set at 0.05 was used for group comparisons. Receiver-Operating Characteristics (ROC) and Area Under the Curve (AUC) were determined to gauge diagnostic capacity.

## 3 Results

**Figure 1:**
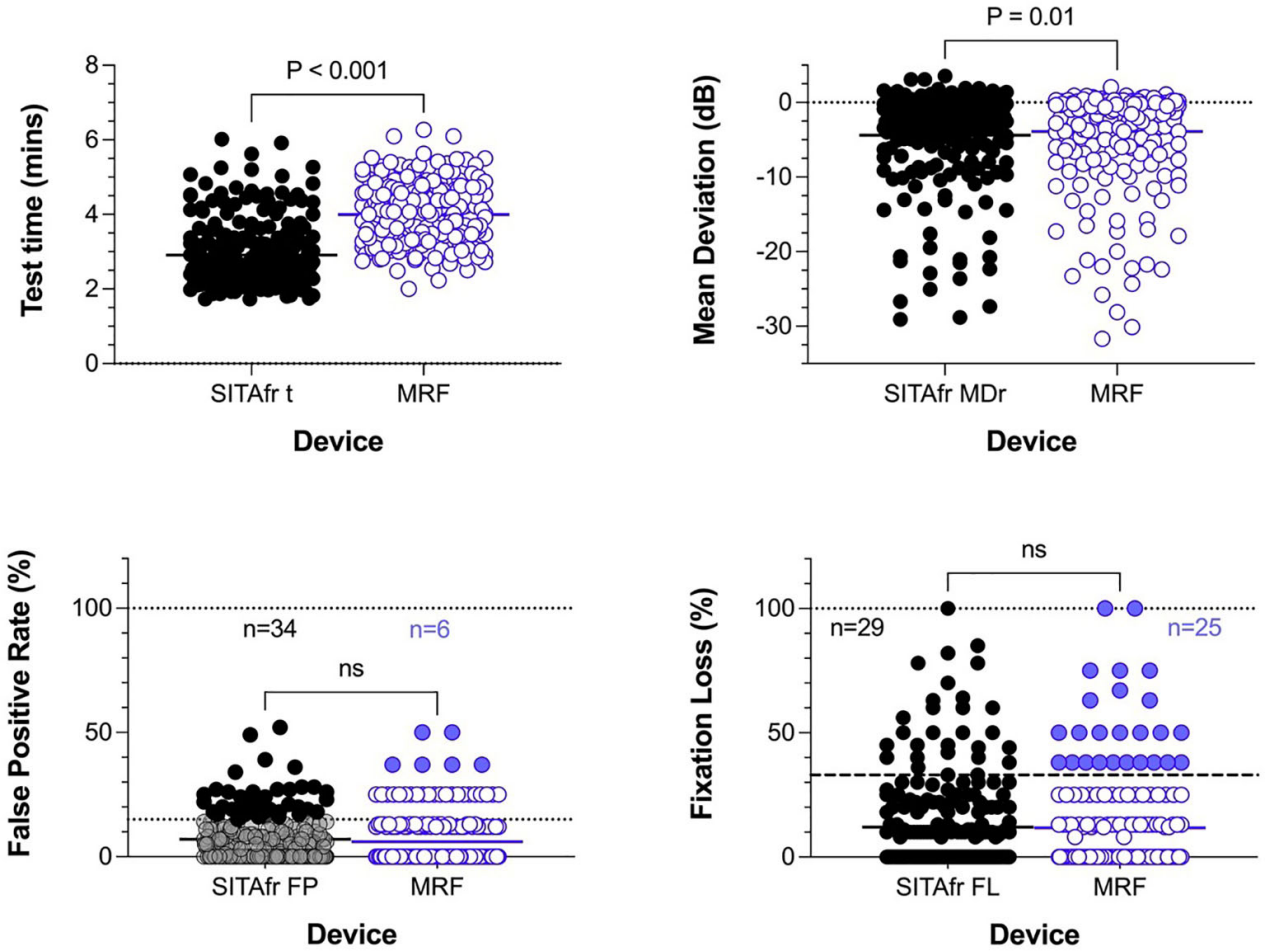
Cohort distributions for test time, Mean Deviation, and reliability indices for False positive (%) and Fixation loss (%). The solid horizontal bar identifies the cohort mean and the p-value shows the outcome of a paired t-test. The lower two panels plot reliability outcomes; these do not differ significantly between tests. The dotted horizontal line in the lower left panel identifies 15% False positives showing that 34 (15%) participants gave unreliable HFA outcomes (black circles) using this criterion. N refer to the number of cases failing at the recommended criterion. The recommended criterion for MRF is a 33% False Positive Rate which 6 participants exceeded (blue circles). Likewise, fixation loss measured using a blind spot monitor finds cohort means that were not significantly different and 29 (12.5%) and 25 (11%) participants exceeded the 33% criterion.

The clinical workup of our 232 participants identified 142 cases of stable glaucoma (60%), 50 glaucoma suspects (22%) and 40 normal eyes (18%). Patients were aged 21 to 92 years (mean of 66.3, standard deviation ± 16.1) with 114 identifying as male (49%), 116 female 50%) and 2 as other (0.9%). Of the 232 eyes analysed, 106 (46%) returned a SITAfr mean deviation in the normal range (MD > -2 dB), 73 (31%) had MD consistent with a mild visual field loss (-2dB to -6dB); 29 (12%) moderate loss (-6 to -12dB); 11 (5%) advanced loss (-12 to -20dB); and 13 (6%) had severe loss (MD ≤ -20dB). All participants were successful in completing testing on MRF-web. The reliability index False positive rate and fixation loss, for the MRF-web and HFA SITAfr did not significantly differ between the two methods (**Figure 1**). MRF test time is approximately 1 minute longer than HFA SITAfr (**Figure 1**, 4.0 ± 0.82 vs 2.9 ± 0.88 mins, p<0.001) which reflects the extra time needed for fixation changes when testing peripheral locations (>18°) of the visual field with MRF-web.

**Figure 2:**
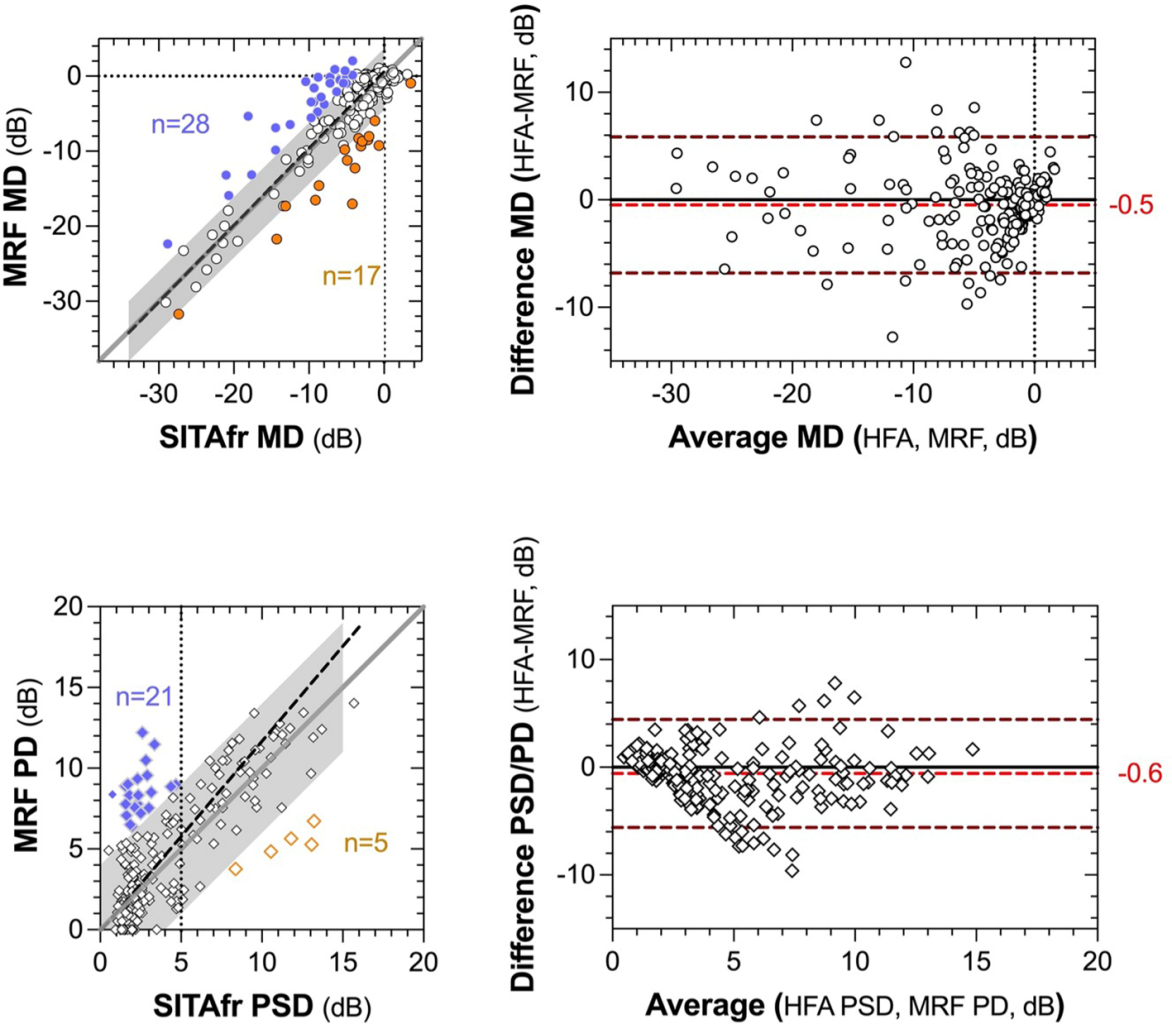
Concordance between global indices (MD and PSD) returned by MRF-web and SITAfr plotted as correlations (left panels) and Bland-Altman analysis (right panels). The left panels present MD in the upper plot and PSD, PD in the lower plot. The grey region identifies a zone of ±4 dB from the unity line (dark grey diagonal). The dashed black line shows the Deming regression to the data (MD slope, 1.0; PSD slope, 1.2). The coloured data identify >4 dB overestimates for MRF (blue) and SITAfr (orange). The vertical dashed line in the lower panel identifies 5 dB PSD for SITAfr. The right panels present the Bland-Altman outcomes for the same data as the left panels. The red dashed line and alphanumerics indicate the bias between test outcomes and the upper and lower dashed lines define the 95% Limits of Agreement in each plot.

The average MRF-web MD is 0.49 dB smaller than HFA SITAfr (-4.4 ± 6.26 vs -3.9 ± 6.38, p<0.01, **Figure 1**). **Figure 2** shows the concordance between MRF and SITAfr MDs where it is apparent that one reason for the difference between devices is that 28 MRF outcomes underestimate the SITAfr MD by more than 4 dB (blue symbols in **Figure 2**), especially at low MD values (≥ -10 dB). This compares with 17 underestimates of the MRF MD by SITAfr (orange symbols in **Figure 2**) which are also found at low MD values. Despite this scatter in 19% (28 + 17 = 45 of 232) of data having early or normal MD, the concordance between devices is good, with a Deming regression of y=1.0*x+0.58 and an ICC = 0.87 [0.84 to 0.90]. **Figure 2** right panels support the presence of high concordance by finding a small bias in the Bland-Altman analysis with the HFA returning -0.5 dB more negative MD on average and 95% LoA of -6.8 to 5.8 dB.

MRF PD is on average 0.7 dB larger than HFA (4.6 ± 3.72 vs 3.9 ± 3.28, p<0.001) due to the 21 (9%) MRF PD values that overestimate HFA PSD by more than 4 dB at low PSD values (<5 dB, **Figure 2**). As could be expected from this finding the PD returns a lesser ICC = 0.73 [0.67 to 0.79] than found for the MD and the Bland-Altman shows a bias of -0.58 dB with 95% LoA of -5.6 to 4.4 dB. The HFA SITAfr vs MRF MD 95% CI for the cohort in the metropolitan Melbourne location (-6.03dB, 4.46dB) was comparable to that from the rural Dubbo location (-7.70dB, 7.77dB).

**Figure 3:**
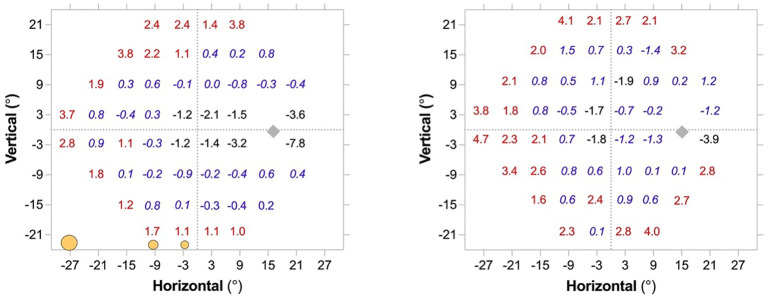
Average pointwise difference (MRF-HFA) for locations in the 24-2 test grid for a subset of participants (n=90) tested at one of our locations. The left panel shows the pointwise difference for 38 participants who returned normal MD (≥ -2 dB) with SITAfr. The Right panel shows the pointwise difference for 52 participants who returned an abnormal MD (< -2 dB) with SITAfr. The RED colour identifies locations where MRF sensitivity was significantly higher (P<0.05), the BLACK negative values identify locations where HFA sensitivity was significantly higher (P<0.05) whereas the blue italicised values are not significantly removed from zero. The general trend is consistent with an increase in spot size at peripheral locations as shown by the annotation of circles on the bottom of the left panel.

The MRF has been designed so that spot size increases with eccentricity. We analysed the pointwise difference between SITAfr and MRF outcomes in an eccentricity-related manner in 38 participants returning normal SITAfr outcomes (MD>-2 dB) and 52 participants with stable glaucoma. These are shown in **Figure 3**. The spot scaling of the MRF returns higher thresholds (by 2-5 dB) at peripheral locations and lower thresholds in the macula.

### 3.1 Diagnostic capacity

**Figure 4:**
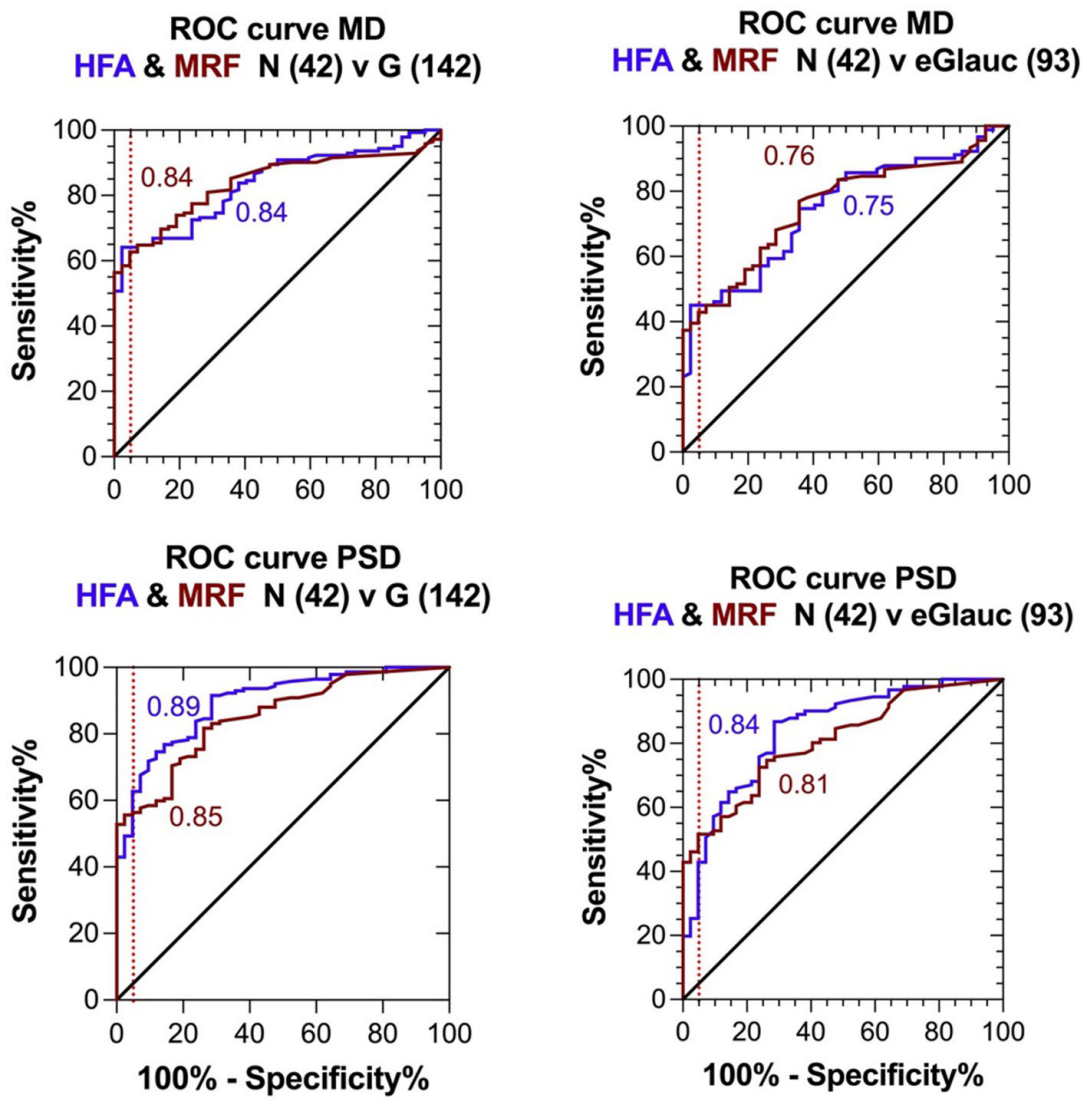
Receiver Operator Characteristics (ROC) for the clinical diagnosis (Normal or Glaucoma) of participants based on extensive clinical evaluation compared with visual field outcomes returned by the MRF and SITAfr tests. Left panels: entire glaucoma group, MD presented in the top figure and PD, PSD in the bottom figure. Right panels: early glaucoma group (SITAfr MD ≥ -6 dB), MD presented in the top figure and PD, PSD in the bottom figure.

The higher peripheral thresholds will allow clinicians to monitor defects for longer because they provide a larger dynamic range. They also produce less variability in the threshold estimate facilitating the detection of change earlier (19, 22, 23). To compare the diagnostic capacity of the MRF and HFA, the Receiver-Operating Characteristic was determined in identifying normal and glaucoma groups that have been diagnosed by clinical methods that combine clinical examination in addition to Humphrey visual field testing. This is shown in **Figure 4** for the entire cohort of participants (n=42 normal, 142 glaucoma) as well as for a subgroup of participants having early glaucoma (MD >-6 dB). The left panels confirm that both tests return similar AUC for both the MD (0.84 ± 0.03 MRF vs 0.84 ± 0.04 HFA) and PSD (0.85 ± 0.03 MRF vs 0.89 ± 0.03 HFA) when considered over the entire group. The right panels show a similar analysis for the early glaucoma group (N=93 eGlauc).

**Figure 5:**
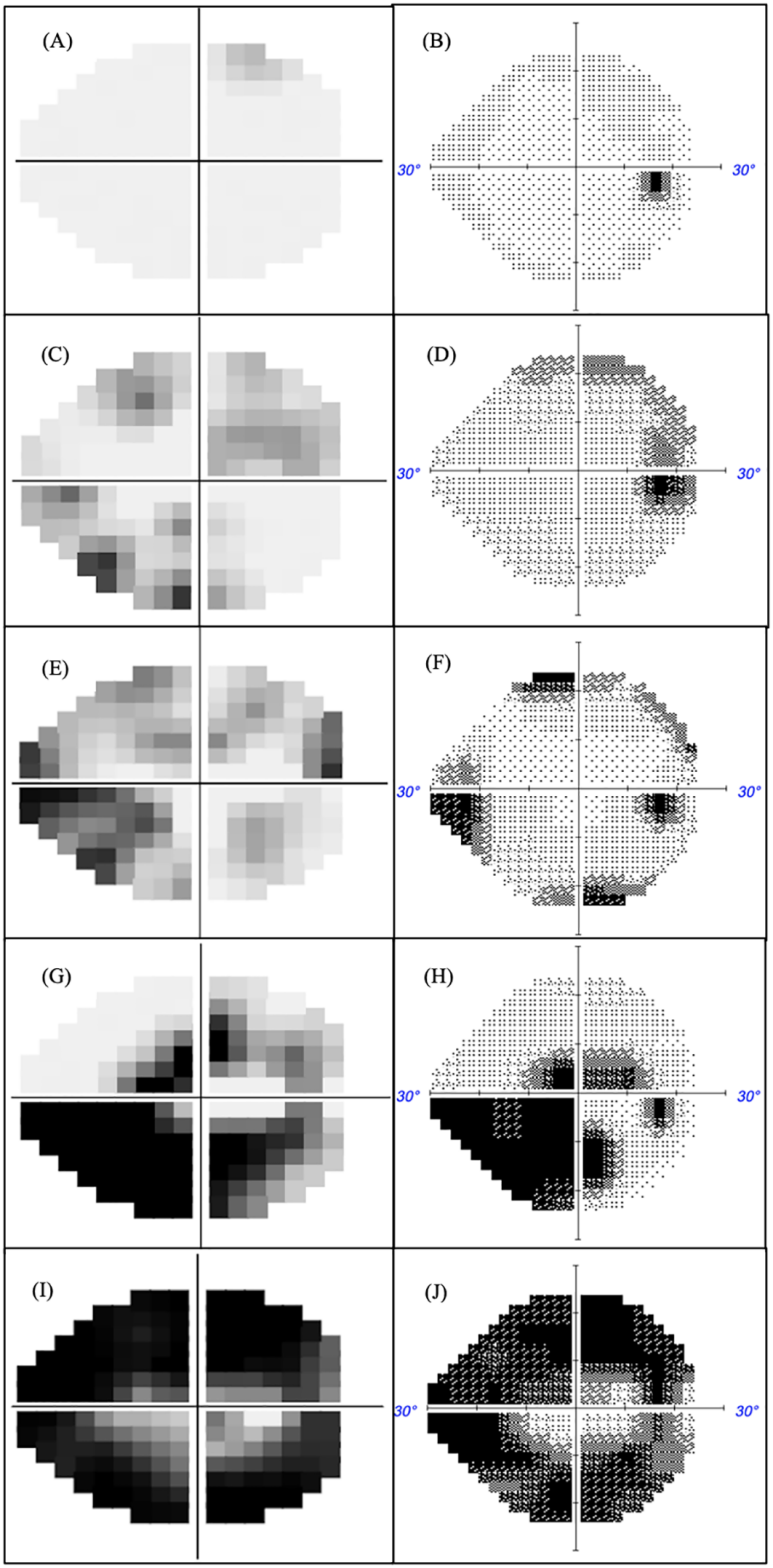
MRF (left) and HFA (right) were measured in the same eye with glaucoma severity increasing in terms of MD. Normal fields MD >-2 **(A, B)**, mild field loss -2<MD>-6 **(C, D)**, moderate field loss MD<-6 **(E, F)**, advanced field loss MD<-12 **(G, H)** and severe field loss MD<-20 **(I, J)**.

The optimal MD criterion for clinical applications of MRF is -1.5 dB which yields a sensitivity of 62% (± 4%) at a 95% specificity (**Figure 4** dashed line). On the other hand, a HFA criterion of -2.5 dB is needed to yield a sensitivity of 64% (± 4%) at a 95% specificity. Likewise, if one considers the bottom right panel for PSD, PD in cases of early glaucoma, one can see that the MRF PD has a significantly higher sensitivity (46% to 52%, criterion 3.2 dB) for early glaucoma at a 95% specificity (red dashed vertical line) than does the HFA PSD (25% to 43%, criterion 3.1 dB, P<0.05). This suggests that clinicians need to consider both the MD and PD when diagnosing early glaucoma using the criteria levels given above. **Figure 5** shows exemplary results for participants having normal through to severe visual field loss.

## 4 Discussion

MRF software, previously as an iPad app, has been found to show strong concordance to HFA SITA-Standard (ICC 0.93 for MD and 0.86 for PD), furthermore, the MRF was found to have a test-retest reliability comparable to HFA (8). The present study demonstrates that a device-independent web-browser based MRF-web software shows a high level of concordance with HFA SITAfr outcomes in terms of global indices (ICC 0.87 for MD and 0.73 for PD) and local pointwise values. In addition, test time and the reliability indices for false positive rate and fixation losses did not significantly differ between the web-MRF and HFA SITAfr. Although the MRF-web on average was found to take 1.2 minutes longer for testing than HFA SITAfr, the additional time reflects the time that was needed for fixation changes when testing peripheral locations. The threshold for MRF-web is higher in the far peripheral locations compared to HFA SITAfr (**Figure 3**), largely due to the increased size of the spot. The higher peripheral thresholds will allow clinicians to monitor defects for longer as they provide a larger dynamic range. They also produce less variability in the threshold estimate facilitating the detection of change earlier (19, 22, 23). This is apparent in the ROC analysis for our group of patients.

Our study shows that MRF-web can be a viable alternative to HFA SITAfr, with the benefit of accessibility, and reduced cost, which can particularly benefit rural locations and underdeveloped areas. In addition, the MRF-web could also potentially serve as a reliable home monitoring tool for glaucoma patients or suspects as highlighted in a previous study by Harris et al. who allowed forty healthy young participants with normal visual function to conduct the MRF-web in their homes, a day after doing a session in a controlled clinical environment (16). In that study, home PC monitors for each participant were of different sizes and configurations, and the screens were calibrated with methods similar to those employed in this study. The research group assessed the consistency of results across the 54 target locations of the 24-2 test pattern. Based on their findings, both environments returned an average consistency of less than 1dB for most locations and gave comparable outcomes. Although the study was limited by a low number of normal participants, Harris et al. demonstrated a promising potential for MRF-web, that it will return findings in controlled settings similar to those when performed at home (16). The consistency in results between the two test environments used by Harris et al. accords with the notion that although light levels vary across all screens (including those of participants at home), the variations are in synchrony for spot and background. For instance, a darker background is associated with a darker spot, therefore contrast is not affected to any appreciable degree (24). Nevertheless, the Harris et al. study did not consider the correlation between MRF-web and the HFA in patients with glaucoma (16). We note that our study is the first to analyse the quantitative correlation of the MRF-web compared to HFA SITA-Faster in glaucoma patients using commercial computers and screens.

There are certain limitations to our study. Firstly, this study is a single cross-sectional study and has not assessed the test-retest variability of the two devices. Our previous work using MRF iPad devices however showed the test-retest variability for MRF is comparable with HFA SITA standard (15). Secondly, our study assessed the comparability of the MRF-web and HFA on two screen sizes and configurations. It will be beneficial to consider the repeatability of MRF-web on a diverse range of computer screens. Additionally, while all patients in our study were able to complete the MRF-web visual field test online, as most glaucoma patients are of older age, there may be a small number of patients who might struggle to utilize this technology (25). Further studies with larger sample sizes and diverse PC equipment may be required to assess outcomes generated by the software and optimize the user experience for older patients.

The promising findings from this study suggest the MRF-web provides an inexpensive, portable, and highly accessible option for visual field testing, which can pave the way for assessing visual fields in settings where standard automated perimetry is not easily accessible including the home environment.

## Data Availability

The raw data supporting the conclusions of this article will be made available upon request from the corresponding author.
